# Metabolic Cost of Dendritic Ca^2+^ Action Potentials in Layer 5 Pyramidal Neurons

**DOI:** 10.3389/fnins.2019.01221

**Published:** 2019-11-12

**Authors:** Guosheng Yi, Yaqin Fan, Jiang Wang

**Affiliations:** School of Electrical and Information Engineering, Tianjin University, Tianjin, China

**Keywords:** Ca^2+^ action potential, energy cost, dendrites, computational model, pyramidal neuron

## Abstract

Pyramidal neurons consume most signaling-related energy to generate action potentials (APs) and perform synaptic integration. Dendritic Ca^2+^ spike is an important integration mechanism for coupling inputs from different cortical layers. Our objective was to quantify the metabolic energy associated with the generation of Ca^2+^ APs in the dendrites. We used morphology-based computational models to simulate the dendritic Ca^2+^ spikes in layer 5 pyramidal neurons. We calculated the energy cost by converting Ca^2+^ influx into the number of ATP required to restore and maintain the homeostasis of intracellular Ca^2+^ concentrations. We quantified the effects of synaptic inputs, dendritic voltage, back-propagating Na^+^ spikes, and Ca^2+^ inactivation on Ca^2+^ spike cost. We showed that much more ATP molecules were required for reversing Ca^2+^ influx in the dendrites than for Na^+^ ion pumping in the soma during a Ca^2+^ AP. Increasing synaptic input increased the rate of dendritic depolarization and underlying Ca^2+^ influx, resulting in higher ATP consumption. Depolarizing dendritic voltage resulted in the inactivation of Ca^2+^ channels and reduced the ATP cost, while dendritic hyperpolarization increased the spike cost by de-inactivating Ca^2+^ channels. A back-propagating Na^+^ AP initiated in the soma increased Ca^2+^ spike cost in the apical dendrite when it coincided with a synaptic input within a time window of several milliseconds. Increasing Ca^2+^ inactivation rate reduced Ca^2+^ spike cost, while slowing Ca^2+^ inactivation increased the spike cost. The results revealed that the energy demand of a Ca^2+^ AP was dynamically dependent on the state of dendritic activity. These findings were important for predicting the energy budget for signaling in pyramidal cells, interpreting functional imaging data, and designing energy-efficient neuromorphic devices.

## Introduction

The brain has powerful capacity of information processing, which makes a substantial contribution to the body’s energy consumption. The limited availability of energetic resources constrains the processing power, size, and architecture of the brain (Attwell and Laughlin, 2001; Laughlin, 2001; Lennie, 2003; Attwell and Gibb, 2005), which determines both the evolution of brain circuitry and generation of functional imaging signals based on related metabolic mechanisms (Howarth et al., 2012). Most brain energy is used on electrical signaling, including action potential (AP) generation, maintaining resting potentials, dendritic integration, and synaptic transmission (Attwell and Laughlin, 2001; Harris et al., 2012; Howarth et al., 2012). The metabolic energy used for neural signaling constrains the flow of information within and between cells, which is dependent on neuron type (Sengupta et al., 2010), excitation/inhibition balance (Sengupta et al., 2013; Yu et al., 2018), coding strategy (Yang et al., 2017; Yu and Yu, 2017), and system size (Yu and Liu, 2014; Yu et al., 2016). Determining the signaling-related energy of different cell types has important implications for the brain’s evolution and function, which also offers considerable insights into the interpretation of functional imaging signals (Howarth et al., 2012; Magistretti and Allaman, 2015) and provides inspirations for engineers to mimic neural circuits to design neuromorphic devices (Cruz-Albrecht et al., 2012; Sengutpa and Stemmler, 2014).

Pyramidal neurons are the main integrators in the cortical column (Spruston, 2008). Their unique dendrites span all cortical layers (Binzegger et al., 2004), which have powerful ability to process excitatory and inhibitory signals (Magee, 2000; Spruston, 2008; Stuart and Spruston, 2015). The excitatory synaptic inputs produce local depolarization in their dendrites. Once the depolarization reaches the threshold for activation of voltage-dependent Ca^2+^ channels, a Ca^2+^ AP is generated in the apical dendrites (Magee, 2000; Stuart and Spruston, 2015). Such threshold-dependent, regenerative response provides a cellular mechanism in pyramidal cells for coupling inputs arriving at different cortical layers (Larkum et al., 1999; Larkum, 2013). Particularly, the Ca^2+^ AP produces long (up to 50 ms) plateau-type depolarization in the distal dendrites, which spreads to the soma/axon to activate voltage-dependent Na^+^ channels, resulting in high-frequency bursting of Na^+^ APs (Larkum, 2013). A back-propagating AP (bAP) initiated in the soma/axon can facilitate the initiation of Ca^2+^ APs if it coincides with synaptic inputs in a short time window, which is also referred to as a backpropagation activated Ca^2+^ (BAC) firing (Larkum et al., 1999; Larkum, 2013). As an active dendritic integration, the Ca^2+^ spike plays a crucial role in neural computation, network dynamics, and cortical processing (Magee, 2000; Spruston, 2008; Larkum, 2013; Stuart and Spruston, 2015).

The electrical signaling within neurons is sustained and propagated via ionic currents through their membranes. The different ion concentrations on the inside and the outside of a cell create electrochemical gradients. They are the major driving forces of neural activity, which store the potential energy and create a cellular battery for each ion (Izhikevich, 2007). The generation of an AP runs down these ion gradients. To maintain signaling, the intracellular ion concentrations need to be actively restored by relevant pumps against ion gradients at the end of each spike, which expend metabolic energy from ATP hydrolysis (Attwell and Laughlin, 2001; Howarth et al., 2012). By converting Na^+^ influx into ATP values, the AP energy cost in the soma/axon was determined in different cell types. It is shown that reversing the Na^+^ influx producing APs makes a significant contribution to the overall usage of signaling-related energy in the brain (Attwell and Laughlin, 2001; Harris et al., 2012; Howarth et al., 2012). The AP shape (Sengupta et al., 2010), Na^+^ channel activation and inactivation (Hasenstaub et al., 2010), membrane voltage (Hallermann et al., 2012), dendritic properties (Yi et al., 2017), body temperature (Yu et al., 2012), and stimulus intensity (Yi et al., 2015) all contributed to the energy cost of a Na^+^ spike. However, it is largely unknown how Ca^2+^ APs in the dendrites contribute to the metabolic demand of neural computation. We addressed this question through the simulation of morphology-based biophysical models of layer 5 (L5) pyramidal cells.

The objective of this study was to estimate the energy consumption associated with the dendritic Ca^2+^ APs. We used computational models of L5 pyramidal cells to simulate a Ca^2+^ spike initiated in the apical dendrites. Our results revealed that the ATP cost of a Ca^2+^ AP was dependent on the state of dendritic activity, which was determined by the synaptic inputs, membrane voltage, bAP, and Ca^2+^ inactivation. These results suggested that the pyramidal neurons dynamically adjust their energy demand according to dendritic responses.

## Materials and Methods

### Computational Model

**FIGURE 1:**
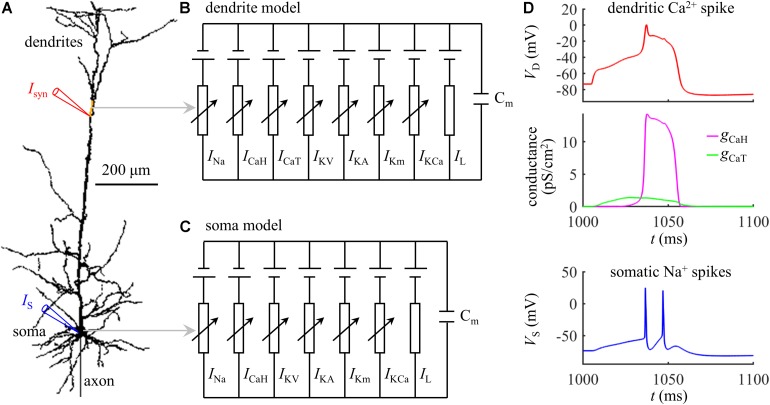
**(A)** Schematic of the computational model of a L5 pyramidal neuron, which included a soma, an axon, and a dendritic tree. The yellow dendrite indicated the active region with increased Ca^2+^ channel densities. **(B)** Dendrite model. **(C)** Soma model. **(D)** Time courses of a dendritic Ca^2+^ spike, Ca^2+^ conductances *g*_*CaH*_ and *g*_*CaT*_, and somatic Na^+^ spikes (*I*_*syn*_ = 1.47 nA and *I*_*S*_ = 0 nA). The site of distal recording/injection was indicated by a red triangle, and the site of somatic recording/injection was indicated by a blue triangle.

The simulations were based on a biologically realistic model of a L5 pyramidal cell, which was available for public download at https://senselab.med.yale.edu/modeldb/ShowModel.cshtml?model=83344. As shown in Figure 1A, the model included a soma, a dendritic tree, and a myelinated axon. The three-dimensional morphology was reconstructed from a pyramidal cell in the somatosensory cortex of a Wistar rat. The passive parameters and active currents followed Schaefer et al. (2003), which were previously validated to reproduce the experimentally documented Ca^2+^ APs in the apical dendrites of neocortical pyramidal neurons. We implemented the computational model in NEURON simulation environment (Hines and Carnevale, 2001), which were solved with a time step of 0.025 ms.

Experimental recording identified that the Ca^2+^ spikes were initiated at the main bifurcation of the apical dendrites (Larkum et al., 1999; Larkum, 2013). To account for BAC firing, Schaefer et al. (2003) introduced an active region with increased Ca^2+^ channel densities to the Ca^2+^ spike initiation zone, which was indicated by a yellow dendrite in Figure 1A. A schematic representation of the membrane model in the active dendrite was shown in Figure 1B, which included fast Na^+^ (*I*_*Na*_), high-voltage activated Ca^2+^ (*I*_CaH_), low-threshold T-type Ca^2+^ (*I*_CaT_), fast K^+^ (*I*_KV_), A-type K^+^ (*I*_KA_), slow non-inactivating K^+^ (*I*_Km_), Ca^2+^-dependent K^+^ (*I*_KCa_), and passive leakage (*I*_*L*_) currents. The channel densities in the active dendrite were as follows (in pS/cm^2^): ${\bar{g}}_{\text{Na}}=$27, ${\bar{g}}_{\text{KV}}=$30, ${\bar{g}}_{\text{KA}}=$300, ${\bar{g}}_{\text{Km}}=$0.1, ${\bar{g}}_{\text{KCa}}=$3.25, ${\bar{g}}_{\text{CaH}}=$4.5, and ${\bar{g}}_{\text{CaT}}=$5. In non-active dendrites, only Ca^2+^ channel densities were different, which were ${\bar{g}}_{\text{CaH}}=$1.5 and ${\bar{g}}_{\text{CaT}}=$0. The soma included *I*_*Na*_, *I*_CaH_, *I*_KV_, *I*_KA_, *I*_Km_, *I*_KCa_, and *I*_*L*_ (Figure 1C). The channel densities in the soma were ${\bar{g}}_{\text{Na}}=$54, ${\bar{g}}_{\text{KV}}=$ 600, ${\bar{g}}_{\text{KA}}=$600, ${\bar{g}}_{\text{Km}}=$0.2, ${\bar{g}}_{\text{KCa}}=$6.5, and ${\bar{g}}_{\text{CaH}}=$3. The reverse potential for each channel was E_Na_ = 60 mV, E_K_ = −90 mV, E_Ca_ = 140 mV, and E_L_ = −70 mV. Membrane capacitance C_*m*_ was 0.6 μF/cm^2^ in the soma and 1.2 μF/cm^2^ in the dendrites.

### Synaptic Inputs

All the synaptic inputs were injected in the dendrites after an initial period of 1000 ms. Unless otherwise stated, the site of distal recording/injection was at the main bifurcation of the apical dendrites (Figure 1A, red triangle), where was 777.45963 μm from the soma. Following Schaefer et al. (2003), the synaptic input *I*_*syn*_ was modeled with a shape of double exponential, i.e., *f*(*t*) = *e*^−*t*/τ_1_^(1−*e*^−*t*/τ_2_^), which was a validated waveform commonly applied to activate Ca^2+^ APs in experiments (Larkum et al., 1999, 2001). The time constants were τ_1_ = 4 ms and τ_2_ = 0.8 ms (Schaefer et al., 2003), which were used to reproduce the compound excitatory postsynaptic potentials (EPSPs) evoked by layer 1 input. The function *f*(*t*) was adjusted so that the peak of *I*_*syn*_ corresponded to the value referred to as stimulus amplitude.

### Calculation of Energy Cost

Applying an excitatory synaptic input *I*_*syn*_ to the apical dendrites depolarized local membrane voltage. Once dendritic depolarization reached the threshold for triggering voltage-gated Ca^2+^ channels to enter the “open state,” the Ca^2+^ conductances increased dramatically and vast Ca^2+^ ions passively flowed into the dendrites down their concentration gradients, resulting in a Ca^2+^ AP (Figure 1D). At the end of each Ca^2+^ spike, the Ca^2+^ gradient was partly run down. To restore and maintain the homeostasis of intracellular Ca^2+^ levels, the Ca^2+^ pumps, including Ca^2+^-ATPase and Na^+^-Ca^2+^ exchanger, extruded the Ca^2+^ influx against its gradient (Attwell and Laughlin, 2001; Howarth et al., 2012; Yi and Grill, 2019), which consumed ATP molecules.

In the apical dendrites, the low-threshold T-type Ca^2+^ current *I*_CaT_ was calculated by:

(1) $$I_{\text{CaT}}={\bar{g}}_{\text{CaT}}\,m^{2}\,n\,\left(V_{\text{D}}-E_{\text{Ca}}\right)$$

where *m* was the activation gating variable, *n* was the inactivation gating variable, and *V*_*D*_ was the dendritic voltage. The high-voltage activated Ca^2+^ current *I*_CaH_ was calculated by:

(2) $$I_{\text{CaH}}={\bar{g}}_{\text{CaH}}\,h^{2}\,l\,\left(V_{\text{D}}-E_{\text{Ca}}\right)$$

Here *h* was the activation variable, and *l* was the inactivation gating variable. The time and voltage dependency of each gating variable (*x*) was computed by:

(3) $$d\,x/d\,t=\alpha_{x}\,\left(1-x\right)-\beta_{x}\,x=\left(x_{\infty }-x\right)/\tau_{x}$$

where α_*x*_ was the forward rate and β_*x*_ was the backward rate. The steady-state value was *x*_∞_ = α_*x*_/(α_*x*_ + β_*x*_), and the time constant was τ_*x*_ = 1/(α_*x*_ + β_*x*_). For current *I*_CaT_, they were:

(4) $$\left\{ \begin{array}{c} m_{\infty }\,\left(V_{\text{D}}\right)=1/\left\{1+e\,x\,p\,\left[-\left(V_{\text{D}}+55\right)/7.4\right]\right\}\\ \tau_{m}\left(V_{\text{D}}\right)=3+1/\{exp\left[\left(V_{\text{D}}+60\right)/20\right]\\ +exp\left[-\left(V_{\text{D}}+135\right)/15\right]\}\\ n_{\infty }\,\left(V_{\text{D}}\right)=1/\left\{1+e\,x\,p\,\left[\left(V_{\text{D}}+75\right)/5\right]\right\}\\ \tau_{n}\left(V_{\text{D}}\right)=30+1/\{exp\left[\left(V_{\text{D}}+66\right)/4\right]\\ +exp\left[-\left(V_{\text{D}}+425\right)/50\right]\} \end{array}\right.$$

For current *I*_CaH_, the rate functions were given by:

(5) $$\left\{ \begin{array}{c} \alpha_{h}\,\left(V_{\text{D}}\right)=0.055\,\left(V_{\text{D}}+27\right)/\left\{1-e\,x\,p\,\left[-\left(V_{\text{D}}+27\right)/3.8\right]\right\}\\ \beta_{h}\,\left(V_{\text{D}}\right)=0.94\,e\,x\,p\,\left[-\left(V_{\text{D}}+75\right)/17\right]\\ \alpha_{l}\,\left(V_{\text{D}}\right)=0.0001523\,e\,x\,p\,\left[-\left(V_{\text{D}}+13\right)/50\right]\\ \beta_{l}\,\left(V_{\text{D}}\right)=0.0015/\left\{1+e\,x\,p\,\left[-\left(V_{\text{D}}+15\right)/28\right]\right\} \end{array}\right.$$

The distribution of two Ca^2+^ currents was not uniform within a dendrite during Ca^2+^ spikes. The NEURON divided each dendrite into several discrete segments with different membrane areas, and the membrane currents and potentials were distributed uniformly within each segment (Hines and Carnevale, 2001). We recorded Ca^2+^ currents *I*_CaH_ and *I*_CaT_ at the center of the segment, and determined the total Ca^2+^ current *I*_Ca_ by adding *I*_CaH_ to *I*_CaT_. The energy cost in each segment was defined as the amount of ATP expended on Ca^2+^ extrusion during a Ca^2+^ spike (Yi and Grill, 2019). We calculated the Ca^2+^ influx per membrane area by integrating *I*_Ca_ during the 100 ms after the onset of *I*_*syn*_, since the active *I*_CaT_ and *I*_CaH_ took place during this interval (Figure 1D, center). The measured Ca^2+^ influx was converted into ATP demand per area by using the fact that the Ca^2+^-ATPase or Na^+^-Ca^2+^ exchanger hydrolyzed one ATP for every Ca^2+^ extruded (Attwell and Laughlin, 2001; Howarth et al., 2012). We computed the surface area *S*_*dseg*_ of each segment in the NEURON, and the ATP cost in the segment was computed by

(6) $$Q_{\text{Ca}}=S_{\text{dseg}}\,\frac{N_{\text{A}}}{2\,F}\,\int \left[I_{\text{CaT}}\,\left(t\right)+I_{\text{CaH}}\,\left(t\right)\right]\,d\,t$$

where N_*A*_ was Avogadro’s constant and F was Faraday’s constant. The total ATP cost by a single dendrite was determined by summating of ATP consumption in all the segments. Note that one Ca^2+^ ion has two positive charges, we computed the Ca^2+^ entry by integrating Ca^2+^ currents, divided by 2.

The Ca^2+^ spike induced a prolonged depolarization in the apical dendrites, which propagated to the soma/axon to activate a burst of Na^+^ spikes (Figure 1D, bottom). To give a portion of energy budget in L5 pyramidal cells, we calculated the ATP cost on reversing somatic Na^+^ influx evoked by a Ca^2+^ spike. The fast Na^+^ current *I*_*Na*_ in the soma was defined as

(7) $$I_{\text{Na}}={\bar{g}}_{\text{Na}}\,w^{3}\,q\,\left(V_{\text{S}}-E_{\text{Na}}\right).$$

Here *w* was the activation variable, *q* was the inactivation variable, and *V*_*S*_ was the somatic voltage. The kinetics of gating variable *w* and *q* was governed by equation (3), and their rate functions were:

(8) $$\left\{ \begin{array}{c} \alpha_{w}\,\left(V_{\text{S}}\right)=0.182\,\left(V_{\text{S}}+25\right)/\left\{1-e\,x\,p\,\left[-\left(V_{\text{S}}+25\right)/9\right]\right\}\\ \beta_{w}\,\left(V_{\text{S}}\right)=-0.124\,\left(V_{\text{S}}+25\right)/\left\{1-e\,x\,p\,\left[\left(V_{\text{S}}+25\right)/9\right]\right\}\\ \alpha_{q}\,\left(V_{\text{S}}\right)=0.024\,\left(V_{\text{S}}+40\right)/\left\{1-e\,x\,p\,\left[-\left(V_{\text{S}}+40\right)/5\right]\right\}\\ \beta_{q}\,\left(V_{\text{S}}\right)=-0.0091\,\left(V_{\text{S}}+65\right)/\left\{1-e\,x\,p\,\left[\left(V_{\text{S}}+65\right)/5\right]\right\} \end{array}\right.$$

Note that the steady-state value for inactivation gating variable *q* was replaced by:

(9) $$q_{\infty }\,\left(V_{\text{S}}\right)=1/\left\{1+\text{exp}\,\left[\left(V_{\text{S}}+55\right)/6.2\right]\right\}.$$

We recorded *I*_*Na*_ at the center of each somatic segment, and calculated the Na^+^ influx per membrane area by integrating the Na^+^ current curve during 100 ms after the onset of *I*_*syn*_. This integral interval was identical to the calculation of Ca^2+^ influx. Since the Na^+^/K^+^-ATPase hydrolyzed one ATP for every three Na^+^ ions exported (Attwell and Laughlin, 2001; Howarth et al., 2012), we computed the ATP cost of a Na^+^ spike in a somatic segment by

(10) $$Q_{\text{Na}}=S_{\text{sseg}}\,\frac{N_{\text{A}}}{3\,F}\,\int I_{\text{Na}}\,\left(t\right)\,d\,t$$

where *S*_*sseg*_ was the surface area of the segment. The total ATP cost in the soma was the summation of ATP consumption in all the segments.

## Results

### Energy Cost of a Synaptically Evoked Ca^2+^ Spike

We first quantified the ATP cost of a synaptically evoked Ca^2+^ spike. We injected a synaptic input *I*_*syn*_ to the main bifurcation of the apical dendrites (Figure 1A, red triangle), and no current injection was applied to the soma. We calculated the smallest *I*_*syn*_ needed for activation of a Ca^2+^ AP (i.e., threshold), which was *I*_*th*_ = 1.47 nA. We increased *I*_*syn*_ from a subthreshold value (< *I*_*th*_) to suprathreshold (> *I*_*th*_), and recorded the evoked transmembrane voltage and transmembrane currents in the dendrites. We computed the ATP cost on Ca^2+^ ion pumping in the active dendrite at each *I*_*syn*_, and the surface area of this single dendrite was 635.9640 μm^2^.

**FIGURE 2:**
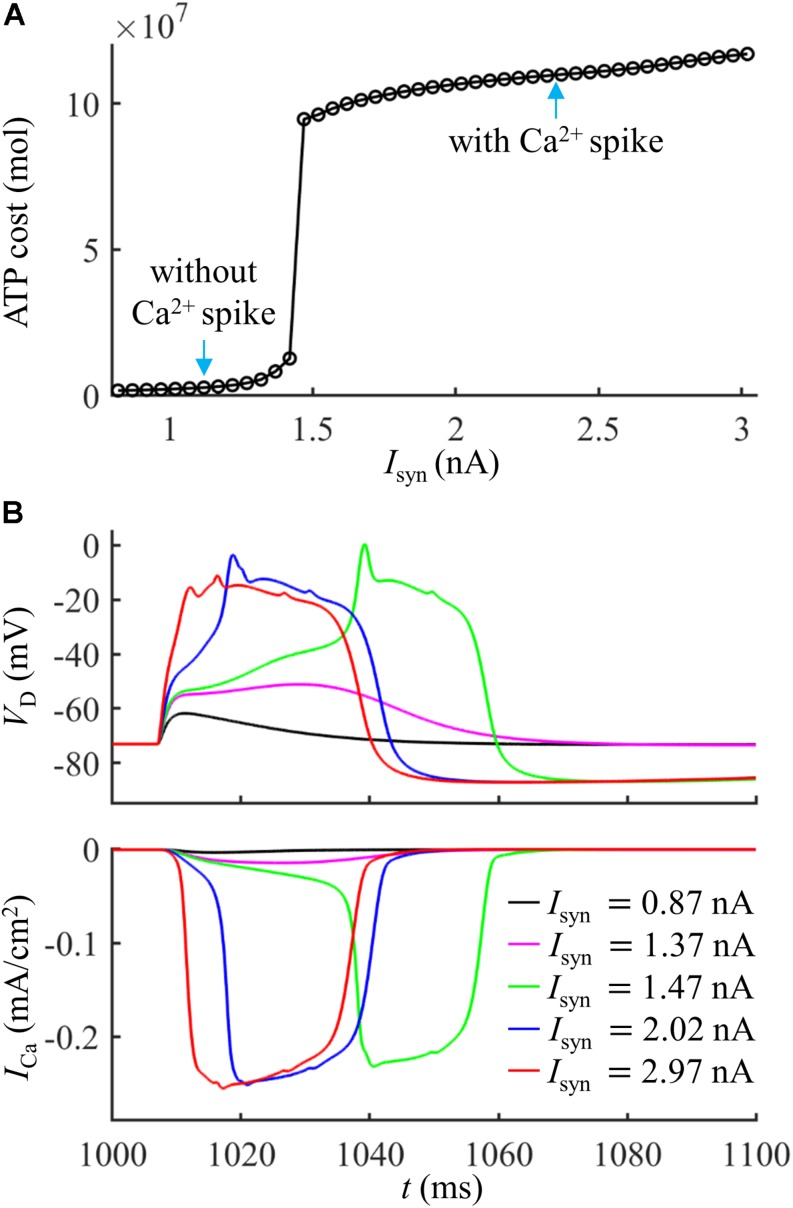
**(A)** ATP cost of a Ca^2+^ spike plotted as a function of *I*_*syn*_. **(B)** Time courses of dendritic voltage *V*_*D*_ and underlying *I*_Ca_ generated at different values of *I*_*syn*_. Somatic injection was *I*_*S*_ = 0 nA.

Extruding Ca^2+^ ions consumed much more ATP during a Ca^2+^ spike than that in the absence of Ca^2+^ spike (Figure 2A). With *I*_*syn*_ ≥ 1.47 nA, the suprathreshold input drove dendritic voltage *V*_*D*_ to reach the threshold for activation of *I*_Ca_ (Figure 2B). This resulted in much larger *I*_Ca_ than that with *I*_*syn*_ < 1.47 nA, thus requiring more ATP for Ca^2+^ extrusion. Increasing suprathreshold *I*_*syn*_ altered the shape of a Ca^2+^ spike, which increased the rate of dendritic depolarization and reduced the amplitude of Ca^2+^ spike. The different spike shapes made the underlying Ca^2+^ currents exhibit different degrees of activation. Specifically, larger depolarization caused the activation gates of Ca^2+^ channels to open faster. Then, *I*_Ca_ reached a higher amplitude before the closing of Ca^2+^ inactivation gate, which resulted in larger Ca^2+^ influx and thus expended more ATP molecules. With *I*_*syn*_ < 1.47 nA, increasing *I*_*syn*_ caused a larger subthreshold depolarization in the dendrite and resulted in higher Ca^2+^ influx. Thus, the ATP cost on Ca^2+^ ion pumping increased with *I*_*syn*_.

**FIGURE 3:**
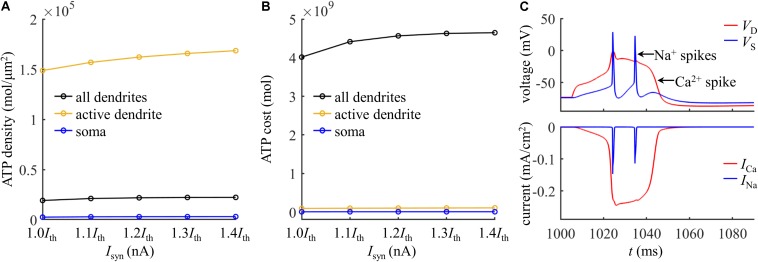
**(A)** ATP cost per unit membrane area during a Ca^2+^ spike. **(B)** Total ATP cost during a Ca^2+^ spike. The synaptic input was injected in the active dendrite, which was *I*_*syn*_ = 1.0*I*_*th*_, 1.1*I*_*th*_, 1.2*I*_*th*_, 1.3*I*_*th*_, and 1.4*I*_*th*_. The ATP cost was, respectively, computed in the soma, active dendrite, and whole dendrites. **(C)** Top panel: time courses of somatic voltage *V*_*S*_ and dendritic voltage *V*_*D*_. Bottom panel: time courses of underlying *I*_*Na*_ and *I*_Ca_ (*I*_*syn*_ = *I*_*th*_ and *I*_*S*_ = 0 nA). *V*_*D*_ and *I*_Ca_ were recorded in the active dendrite, and *V*_*S*_ and *I*_*Na*_ were recorded in the soma.

To give a portion of energy budget in the model neuron, we computed the ATP consumption on Ca^2+^ extrusion in the dendrites and the consumption on Na^+^ ion pumping in the soma. The ATP requirements per unit area in the active dendrite and whole dendrites were both higher as compared to the soma (Figure 3A), which slightly increased with *I*_*syn*_. The surface area of the soma (2352.41291 μm^2^) was about fourfold larger than the active dendrite, but more ATP molecules were required for Ca^2+^ extrusion in this dendrite than for Na^+^ ion pumping in the soma (Figure 3B). To understand different ATP consumption in the dendrites and soma, we compared the Ca^2+^ and Na^+^ spike shapes and underlying currents (Figure 3C). The Ca^2+^ spike caused a prolonged depolarization in the active dendrite, which activated two Na^+^ APs in the soma. The *I*_*Na*_ underlying a Na^+^ spike was smaller and faster than *I*_Ca_ in the active dendrite. Further, the Na^+^/K^+^-ATPase consumed one ATP for every three Na^+^ ions extruded, while the Ca^2+^-ATPase or Na^+^-Ca^2+^ exchanger hydrolyzed one ATP for each Ca^2+^ ion extruded. These factors collectively resulted in a higher requirement of ATP molecules for Ca^2+^ ion pumping in the dendrites than for Na^+^ ion pumping in the soma. This indicated that the generation of Ca^2+^ APs made a significant contribution to the signaling-related energy in L5 pyramidal cells. Note that the Na^+^ influx occurred through fast Na^+^ channels in the dendrites during a Ca^2+^ spike, and pumping these Na^+^ ions out of the dendrites also consumed ATP. However, it contributed less to total ATP consumption as compared to Ca^2+^ extrusion. Similarly, a small number of Ca^2+^ ions entered the soma through *I*_CaH_ during two Na^+^ APs, which consumed ATP to be extruded. Moreover, the Ca^2+^ channel densities were increased artificially in the active dendrite, which also affected the comparison of ATP consumption in the dendrites and soma.

### Effects of Dendritic Membrane Voltage

The activation of Ca^2+^ currents is voltage dependent, and the membrane voltage influences the degree in Ca^2+^ channel availability. We examined the effects of dendritic voltage on the energy cost of a Ca^2+^ spike. We injected a constant current of different amplitudes in the main bifurcation of the apical dendrites to vary local voltage. The duration of constant stimulus was 1000 ms, which was sufficient for all the gating variables of each channel to reach steady states. At the end of 1000 ms constant injection, we applied a synaptic input *I*_*syn*_ to evoke a Ca^2+^ AP in the active dendrite. We used *V*_*init*_ to indicate the value of dendritic voltage at which a Ca^2+^ spike was initiated. Since membrane depolarization reduced Ca^2+^ availability and increased the threshold current, the distal input *I*_*syn*_ was 2.3*I*_*th*_. We quantified the ATP cost of a Ca^2+^ spike in the active dendrite at each *V*_*init*_.

**FIGURE 4:**
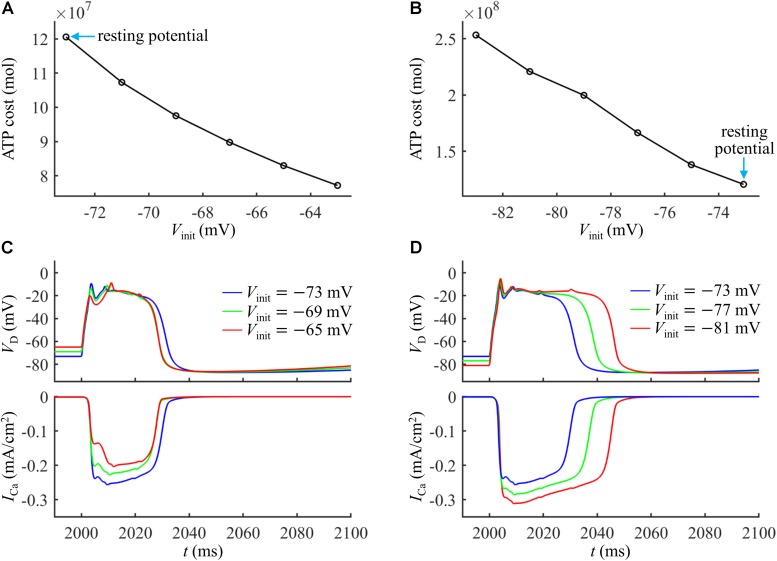
**(A)** Ca^2+^ spike cost in the active dendrite as a function of depolarizing *V*_*init*_. **(B)** Ca^2+^ spike cost in the active dendrite as a function of hyperpolarizing *V*_*init*_. **(C)** Time courses of *V*_*D*_ and underlying *I*_Ca_ recorded at *V*_*init*_ = –65, –69, and –73 mV. **(D)** Time courses of *V*_*D*_ and underlying *I*_Ca_ recorded at *V*_*init*_ = –81, –77, and –73 mV. A constant current of 1000 ms was injected to generate different values of *V*_*init*_ in the active dendrite. At the end of constant stimulus, a synaptic current *I*_*syn*_ was injected to stimulate a Ca^2+^ spike. The injection site of constant and synaptic currents was indicated by a red triangle in Figure 1A. *I*_*syn*_ = 2.3*I*_*th*_ and *I*_*S*_ = 0 nA.

As shown in Figures 4A,B, the ATP cost of a Ca^2+^ spike was reduced with *V*_*init*_. The resting potential in the active dendrite was −73 mV. Depolarizing dendritic voltage (i.e., *V*_*init*_ > −73 mV) resulted in the inactivation of Ca^2+^ channels and reduced channel availability. Then, the *I*_Ca_ underlying each dendritic spike was reduced as *V*_*init*_ increased (Figure 4C), which corresponded to lower Ca^2+^ influx and less ATP expenditure. Hyperpolarizing dendritic voltage (i.e., *V*_*init*_ <−73 mV) de-inactivated Ca^2+^ channels and increased channel availability. This effectively increased the Ca^2+^ current underlying a Ca^2+^ spike and extended the interval of its plateau level (Figure 4D), resulting in more Ca^2+^ influx. Thus, the ATP expenditure on Ca^2+^ extrusion was increased with the hyperpolarization of dendritic voltage. These data suggested that the metabolic cost of a Ca^2+^ spike in the apical dendrite was dynamically dependent on the membrane voltage and Ca^2+^ channel availability.

Note that varying dendritic voltage also affected K^+^ efflux (Yi et al., 2019). The K^+^ currents flowed out of the cell, facilitating the hyperpolarization of dendritic voltage. In particular, the K^+^ efflux overlapped with Ca^2+^ currents at the subthreshold potentials, which made Ca^2+^ influx less efficient in generating dendritic depolarization, thus inflating metabolic energy. In our simulations, the Ca^2+^ inactivation dominated the energy cost of a dendritic spike as *V*_*init*_ was varied, and we did not consider the effects of hyperpolarizing K^+^ currents.

### Effects of Back-Propagating Action Potential

The bAP generated in the soma/axon facilitates the initiation of Ca^2+^ APs when it coincides with distal EPSPs within a time window of several milliseconds (Larkum et al., 1999; Larkum, 2013). We examined the effects of bAP on the metabolic cost of a Ca^2+^ spike.

**FIGURE 5:**
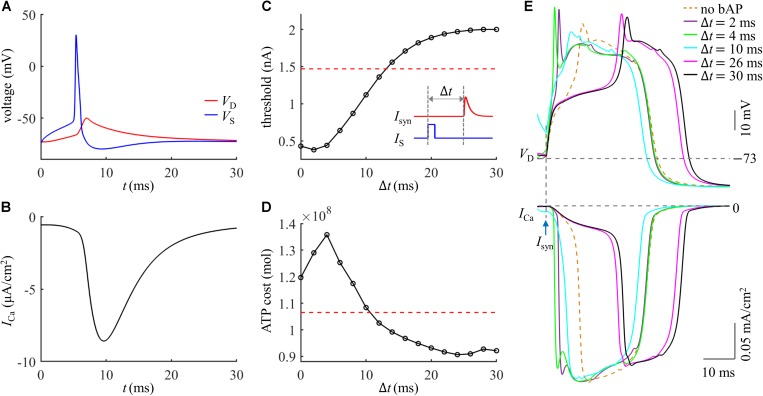
**(A)** Time courses of *V*_*S*_ and *V*_*D*_ in the case of a bAP. **(B)** Time course of Ca^2+^ current *I*_Ca_ during a bAP. **(C)** Threshold for activation of a Ca^2+^ spike in the active dendrite, which was plotted as a function of time interval Δ*t*. Inset: *I*_*syn*_ was injected at Δ*t* after the onset of *I*_*S*_. The red dotted line indicated the threshold without a bAP. **(D)** ATP cost of a Ca^2+^ spike as a function of Δ*t*. The red dotted line indicated the spike cost without a bAP. **(E)** Top panel: superposition of Ca^2+^ spikes generated at different values of Δ*t*. Bottom panel: Ca^2+^ current *I*_Ca_ underlying each dendritic spike. In **(A,B)**, *I*_*syn*_ = 0 nA. In **(D,E)**, *I*_*syn*_ = 2 nA. Somatic input was *I*_*S*_ = 1.9 nA.

We injected a current pulse *I*_*S*_ of 5 ms to evoke a Na^+^ spike in the soma. The amplitude of *I*_*S*_ was 1.9 nA, which was suprathreshold for activation of a Na^+^ AP. The resulting somatic spike backpropagated to the active dendrite, which caused a subthreshold depolarization in the absence of synaptic inputs (Figure 5A) and resulted in a small *I*_Ca_ (Figure 5B). After the onset of *I*_*S*_, we applied a synaptic input *I*_*syn*_ of 2 nA to evoke a Ca^2+^ spike in the active dendrite. As shown in Figure 5C, the threshold of *I*_*syn*_ for eliciting a Ca^2+^ spike reached a minimum when distal input was 2 ms after the onset of Na^+^ AP. The Ca^2+^ spike threshold became higher than that in the absence of a bAP when the time interval Δ*t* was longer than 12 ms. This was consistent with previous experimental recording (Larkum et al., 1999).

We calculated the ATP cost of a Ca^2+^ spike in the active dendrite as the interval Δ*t* of *I*_*syn*_ and *I*_*S*_ increased from 0 ms to 30 ms. Unlike Ca^2+^ spike threshold, the ATP cost of a Ca^2+^ AP reached a maximum when synaptic input *I*_*syn*_ was injected at Δ*t* = 4 ms after the onset of a bAP (Figure 5D). With the delay Δ*t* > 4 ms, the Ca^2+^ spike cost was reduced sharply, which became even lower than that in the absence of a bAP for the delays of over 10 ms. This showed that the energy cost of a Ca^2+^ AP was greatly increased if the dendritic EPSP and bAP were timed so that they coincided within a few milliseconds. But the Ca^2+^ spike cost was slightly reduced if the EPSP followed a bAP 12 ms later.

The inward *I*_Ca_ exhibited different degrees of activation dependent upon the time interval Δ*t* of *I*_*syn*_ and bAP (Figure 5E). With 0 < Δ*t* ≤ 4 ms, the delay was so short that the dendritic depolarization induced low degree of Ca^2+^ channel inactivation. In this case, increasing Δ*t* increased the rate of dendritic depolarization and extended the width of a Ca^2+^ spike, which resulted in more Ca^2+^ influx, thus increasing ATP expenditure from Ca^2+^ channels. With 4 < Δ*t* ≤ 24 ms, the sustained depolarization induced a high degree of Ca^2+^ inactivation, which reduced the availability of Ca^2+^ channels in the dendrite. At these delays, increasing Δ*t* reduced both the rate of dendritic depolarization and the amplitude of inward *I*_Ca_, resulting in less Ca^2+^ influx. Thus, the ATP cost from Ca^2+^ channels was decreased with increasing the delay Δ*t*. With 24 < Δ*t* ≤ 30 ms, the Ca^2+^ inactivation reached a steady state. Increasing Δ*t* only resulted in a small delay of the *I*_Ca_ while produced little effect on the current amplitude, which slightly increased Ca^2+^ influx and corresponding ATP cost.

### Effects of Ca^2+^ Inactivation

Based on above simulations, we inferred that the Ca^2+^ inactivation participated in the metabolic cost of a Ca^2+^ spike. To test this hypothesis, we systematically manipulated the inactivation kinetics of voltage-gated Ca^2+^ channels in the active dendrite. We multiplied Ca^2+^ inactivation rate by a scale factor ranging from 0.2 to 2 at a step of 0.2. We injected a synaptic input *I*_*syn*_ at the main bifurcation of the apical dendrites to evoke a Ca^2+^ spike, and the stimulus amplitude was 1.4*I*_*th*_. We quantified the ATP cost of a Ca^2+^ AP in the active dendrite at each inactivation rate factor.

**FIGURE 6:**
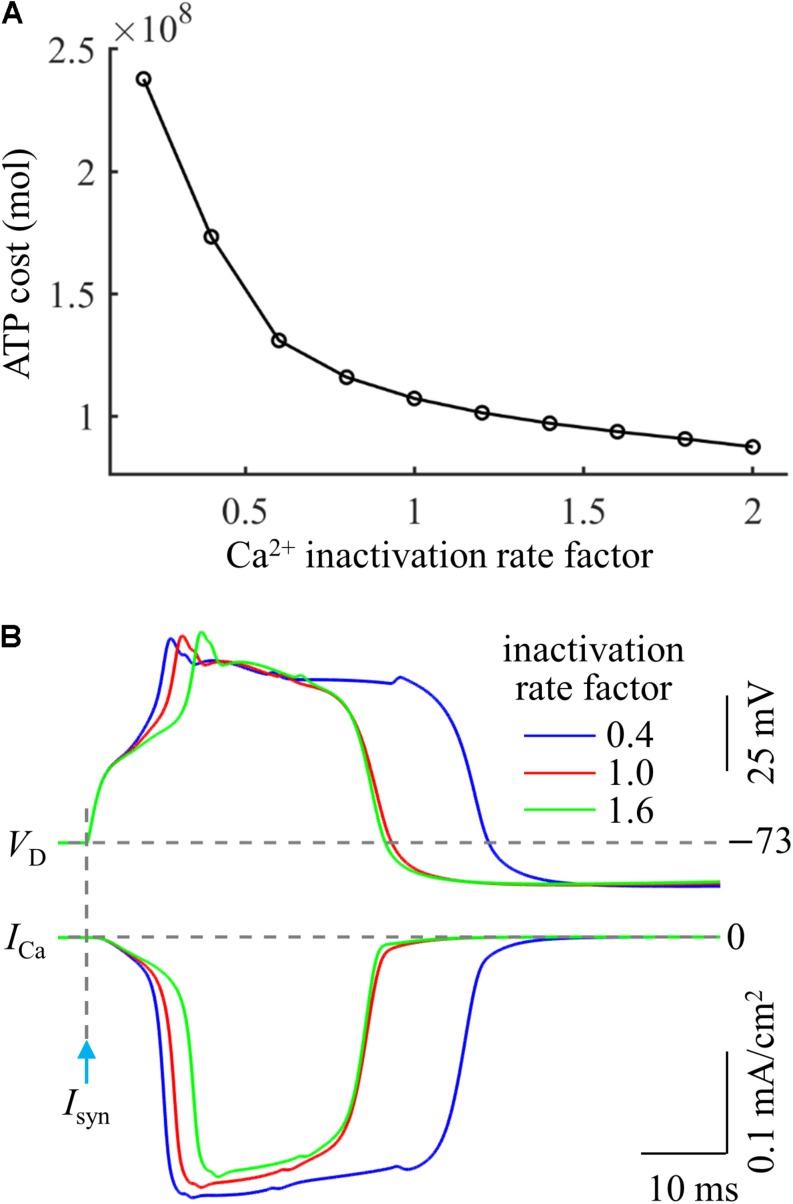
**(A)** ATP cost of a Ca^2+^ spike plotted as a function of Ca^2+^ inactivation rate factor. **(B)** Ca^2+^ spikes and underlying *I*_Ca_ recorded at three inactivation rate factors. *I*_*syn*_ = 1.4*I*_*th*_ and *I*_*S*_ = 0 nA.

As shown in Figure 6A, the Ca^2+^ spike cost was reduced with the speed of Ca^2+^ inactivation. Slowing Ca^2+^ channel inactivation (i.e., factor < 1) increased the rate of dendritic depolarization and extended the width of a Ca^2+^ spike (Figure 6B). This resulted in a larger *I*_Ca_ and increased Ca^2+^ influx, thus more ATP molecules were expended on Ca^2+^ extrusion. On the contrary, increasing the speed of Ca^2+^ channel inactivation (i.e., factor > 1) reduced the rate of dendritic depolarization (Figure 6B), which resulted in a smaller *I*_Ca_, thus decreasing ATP expenditure. These data suggested that the Ca^2+^ inactivation regulated the metabolic cost of a Ca^2+^ spike by altering the spike shape and Ca^2+^ availability.

### Ca^2+^ Spike Cost Evoked by *in vivo* Like Synaptic Inputs

Our above simulations were based on the examination of a single synaptic location. The conditions of synaptic inputs were simply set rather than *in vivo* like, i.e., thousands of synaptic inputs broadly received on the whole dendrites. To generalize our predictions in realistic cases, we examined the energy cost of a Ca^2+^ spike evoked by multiple synaptic inputs.

**FIGURE 7:**
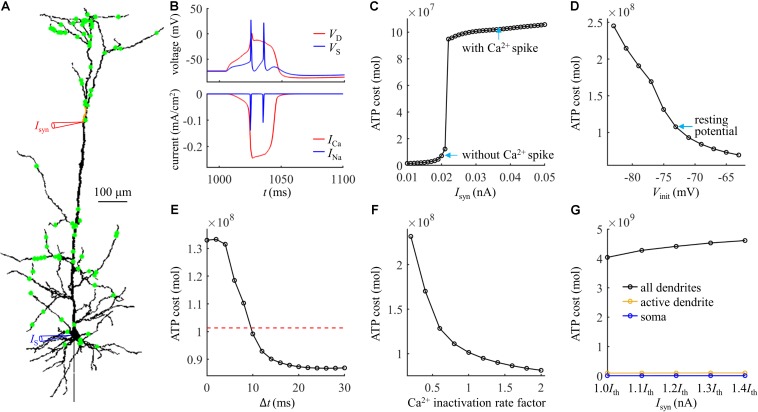
**(A)** Distribution of 100 excitatory inputs (green dots) over the dendrites. Each input *I*_*syn*_ was modeled with a double exponential function *f*(*t*) = *e*^−*t*/τ_1_^(1−*e*^−*t*/τ_2_^). Time constant was τ_1_ = 4 ms and τ_2_ = 0.8 ms. **(B)** Top panel: time courses of somatic voltage *V*_*S*_ and dendritic voltage *V*_*D*_. Bottom panel: time courses of underlying *I*_*Na*_ and *I*_Ca_ (*I*_*syn*_ = *I*_*th*_ and *I*_*S*_ = 0 nA). The threshold *I*_*th*_ for activation of a Ca^2+^ spike was 0.022 nA. *V*_*D*_ and *I*_Ca_ were recorded in the active dendrite, which was indicated by a red triangle in **(A)**. *V*_*S*_ and *I*_*Na*_ were recorded in the soma, which was indicated by a blue triangle in **(A)**. **(C)** ATP cost of a Ca^2+^ spike plotted as a function of *I*_*syn*_ (*I*_*S*_ = 0 nA). **(D)** ATP cost of a Ca^2+^ spike plotted as a function of dendritic voltage. A constant current of 1000 ms was injected to the active dendrite. At the end of 1000 ms constant injection, 100 synaptic inputs were applied to evoke a Ca^2+^ AP. *V*_*init*_ indicated the voltage at which a Ca^2+^ spike was initiated. *I*_*syn*_ = 2.3*I*_*th*_ and *I*_*S*_ = 0 nA. **(E)** ATP cost of a Ca^2+^ spike plotted as a function of the Δ*t*. Here Δ*t* was defined as the interval between the onset of 100 inputs and somatic injection. The red dotted line indicated the ATP cost in the active dendrite without a bAP. *I*_*syn*_ = 1.5*I*_*th*_ and *I*_*S*_ = 1.9 nA. **(F)** ATP cost of a Ca^2+^ spike plotted as a function of Ca^2+^ inactivation rate factor. *I*_*syn*_ = 1.5*I*_*th*_ and *I*_*S*_ = 0 nA. **(G)** ATP cost in the dendrites and soma during a Ca^2+^ spike. *I*_*syn*_ = 1.0*I*_*th*_, 1.1*I*_*th*_, 1.2*I*_*th*_, 1.3*I*_*th*_, and 1.4*I*_*th*_. In **(C–F)**, the ATP cost on Ca^2+^ extrusion was computed in the active dendrite. In **(G)**, the ATP consumption on Ca^2+^ extrusion was computed in the active dendrite and whole dendrites, and the consumption on Na^+^ extrusion was computed in the soma.

We started by applying 100 synaptic inputs to the dendrites. To quantify their distributions, we defined a distance *d*_*syn*_ = 250 μm. 90% of the synaptic inputs were randomly distributed at the distal sites, which were more than *d*_*syn*_ from the soma (Figure 7A). The remaining 10% of the inputs were randomly applied to the proximal sites less than *d*_*syn*_ from the soma. The threshold for activation of a Ca^2+^ spike in the active dendrite was *I*_*th*_ = 0.022 nA. The Ca^2+^ and Na^+^ spike shapes were both similar to those evoked by single synaptic input (Figure 7B). The results in Figures 7C–F showed that the effects of synaptic conductances, dendritic voltage, bAP, and Ca^2+^ inactivation on the ATP cost of a Ca^2+^ spike were all consistent with the simulations based on the examination of a single synaptic location. Under this realistic input condition, more ATP were required for Ca^2+^ ion pumping in the dendrites than for Na^+^ ion pumping in the soma (Figure 7G), which was also in accordance with above predictions.

**FIGURE 8:**
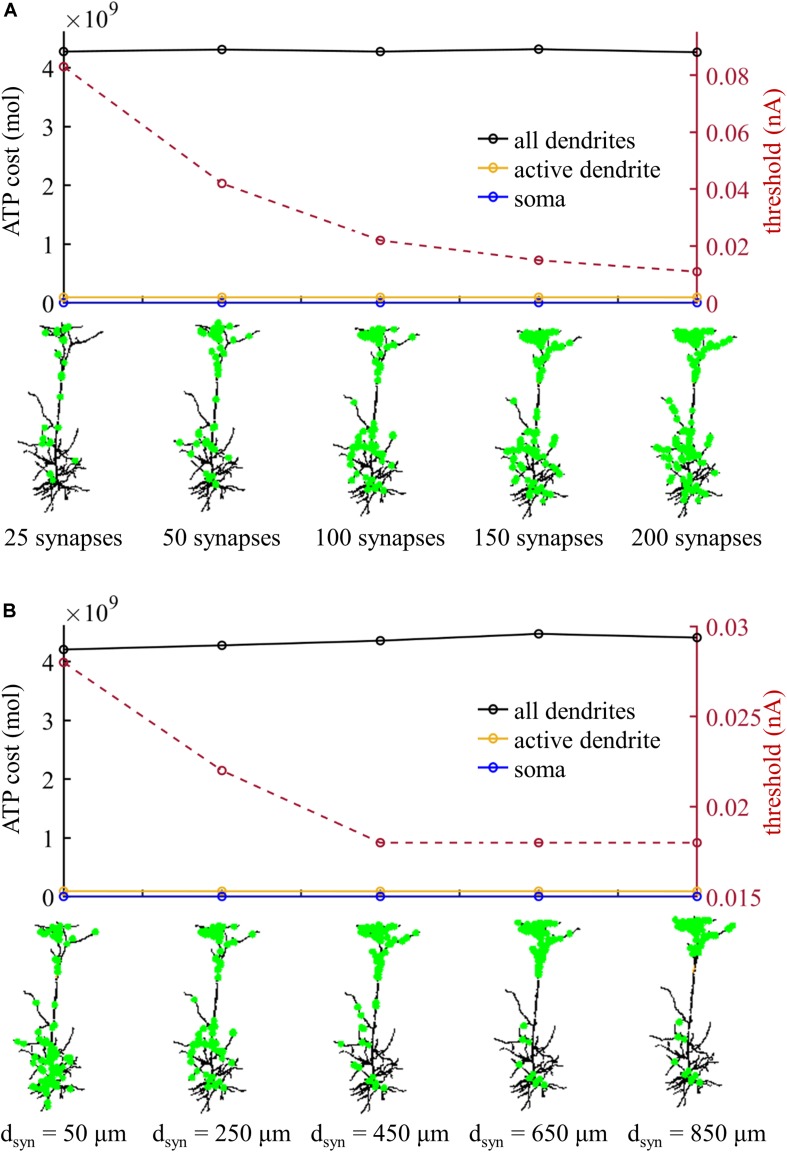
**(A)** ATP cost and Ca^2+^ spike threshold *I*_*th*_ as the number of synaptic inputs increased. 90% of the synaptic inputs were randomly distributed in the sites more than *d*_*syn*_ from the soma, and the distribution of the rest 10% was over the sites less than *d*_*syn*_ from the soma. Here the distance *d*_*syn*_ was 250 μm. **(B)** ATP cost and Ca^2+^ spike threshold *I*_*th*_ as the distance *d*_*syn*_ increased. Here the number of synaptic inputs was 100. When computing the ATP cost, the synaptic inputs were 1.1*I*_*th*_. Somatic input was *I*_*S*_ = 0 nA.

We subsequently examined the effects of the number of synaptic inputs on the ATP cost without changing their distributions over the dendrites. The threshold for activating a Ca^2+^ spike decreased with the number of inputs (Figure 8A). However, the ATP cost on dendritic Ca^2+^ extrusion and somatic Na^+^ ion pumping exhibited little changes as the synaptic number increased from 25 to 200. Particularly, increasing the number of inputs did not alter the comparison of ATP consumption on reversing ion influxes in the dendrites and soma. We also examined the effects of the distributions of 100 synaptic inputs on the ATP cost by altering the value of *d*_*syn*_. As the distance *d*_*syn*_ increased, more synaptic inputs were randomly assigned to the distal dendrites, and lower synaptic conductances were required for activation of a Ca^2+^ spike in the active dendrite (Figure 8B). However, altering the synaptic distributions over the dendrites produced slight effects on the ATP cost in the dendrites and soma.

### Model Predictions With Different Morphologies and Biophysics

**FIGURE 9:**
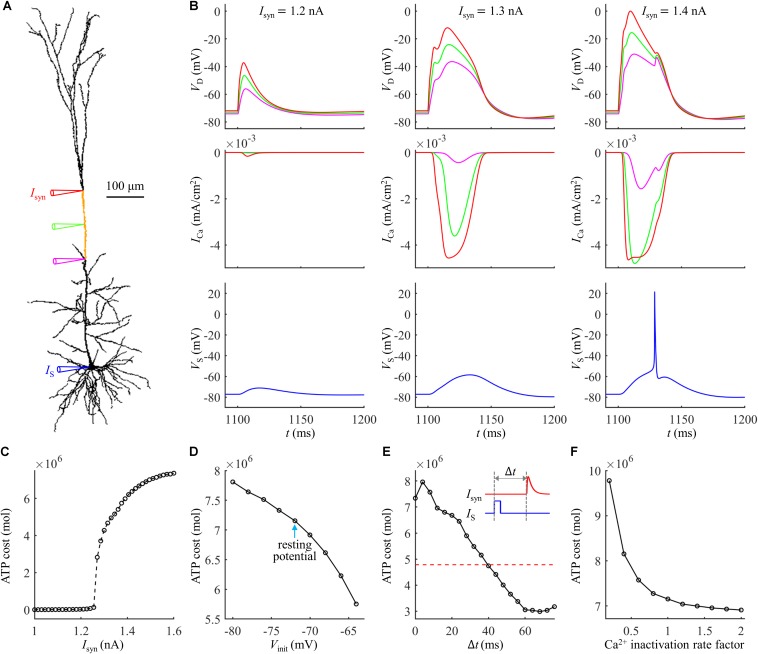
**(A)** Schematic of the computational model of Hay et al. (2011), which was available for public download at https://senselab.med.yale.edu/modeldb/ShowModel.cshtml?model=139653. The site of distal injection was indicated by a red triangle, and the site of somatic injection was indicated by a blue triangle. The ATP cost was computed in the yellow dendrite. The synaptic input *I*_*syn*_ was modeled with a shape of double exponential function. Following Hay et al. (2011), the rise time of *I*_*syn*_ was 0.5 ms and the decay time was 5 ms. **(B)** Time courses of dendritic voltage *V*_*D*_, underlying Ca^2+^ current *I*_Ca_, and somatic voltage *V*_*S*_ at *I*_*syn*_ = 1.2, 1.3, and 1.4 nA. The recording sites were indicated by the triangles of same color in **(A)**. *I*_*S*_ = 0 nA. **(C)** ATP cost of a Ca^2+^ spike plotted as a function of *I*_*syn*_ (*I*_*S*_ = 0 nA). The threshold of synaptic input for activation of a Ca^2+^ spike was *I*_*th*_ = 1.27 nA. **(D)** Ca^2+^ spike cost plotted as a function of dendritic voltage. A constant current of 1000 ms was injected to the yellow dendrite. At the end of 1000 ms constant injection, a synaptic input *I*_*syn*_ was applied to evoke a Ca^2+^ AP. *V*_*init*_ indicated the voltage at which a Ca^2+^ spike was initiated. *I*_*syn*_ = 1.2*I*_*th*_ and *I*_*S*_ = 0 nA. **(E)** ATP cost of a Ca^2+^ spike plotted as a function of the interval Δ*t*. Inset: *I*_*syn*_ was injected at Δ*t* after the onset of somatic injection *I*_*S*_. The red dotted line indicated the spike cost without a bAP. *I*_*S*_ was a 5 ms brief pulse of 1.4 nA. *I*_*syn*_ = 1.31 nA. **(F)** ATP cost of a Ca^2+^ spike plotted as a function of Ca^2+^ inactivation rate factor. *I*_*syn*_ = 1.2*I*_*th*_ and *I*_*S*_ = 0 nA.

The morphologies and biophysics are varied across L5 pyramidal cells. Here we examined our above predictions in a L5 pyramidal model neuron with different morphologies, channel kinetics, and conductances (Figure 9A). The details of the model followed Hay et al. (2011), which captured a wide range of dendritic and perisomatic active properties of L5 pyramidal cells. Especially, it was validated to faithfully replicate the dendritic Ca^2+^ spike and BAC firing recorded *in vitro*. This model did not include an active dendrite with increased Ca^2+^ channel densities.

We injected a synaptic input *I*_*syn*_ at the main bifurcation of the apical dendrites (402.54041 μm from the soma), indicated by a red triangle in Figure 9A. The threshold of synaptic input for activating a Ca^2+^ spike was *I*_*th*_ = 1.27 nA. At *I*_*syn*_ < *I*_*th*_, the dendrites generated passive responses and no Ca^2+^ spike was activated (Figure 9B, left). At *I*_*th*_ ≤ *I*_*syn*_ < 1.36 nA, the dendrites generated a Ca^2+^ spike in the apical dendrite. However, the resulting AP was significantly attenuated as it propagated to the soma/axon, which was unable to activate a Na^+^ spike in the cell body (Figure 9B, center). At *I*_*syn*_ ≥ 1.36 nA, the Ca^2+^ spike activated Na^+^ spikes in the soma (Figure 9B, right).

We repeated above simulations to examine the ATP cost of a Ca^2+^ spike, which was computed in the yellow dendrite shown in Figure 9A. The surface area of this dendrite was 2034.8 μm^2^. The results in Figures 9C–F showed that our above predictions were largely reproduced in this model with different morphologies and biophysics. Here the synaptic inputs, dendritic voltage, bAP, and Ca^2+^ inactivation produced very similar effects on the Ca^2+^ spike cost to those described above. Note that the Ca^2+^ influx underlying a Ca^2+^ spike was much smaller in the model of Hay et al. (2011) compared with the model of Schaefer et al. (2003), and thus the ATP cost on Ca^2+^ extrusion was much lower than that described above.

**FIGURE 10:**
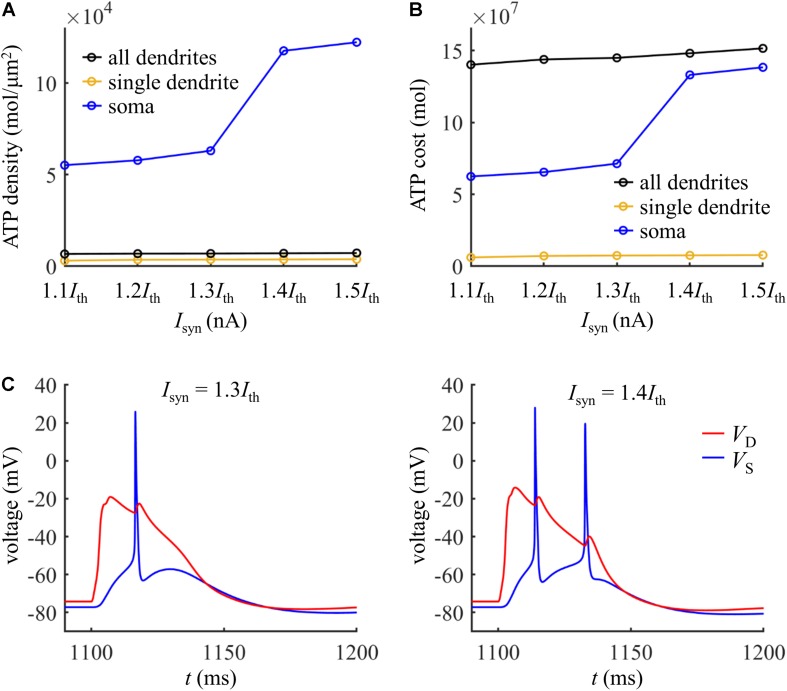
**(A)** ATP cost per unit membrane area during a Ca^2+^ spike. **(B)** Total ATP cost during a Ca^2+^ spike. The synaptic input was injected in the yellow dendrite, which was *I*_*syn*_ = 1.1*I*_*th*_, 1.2*I*_*th*_, 1.3*I*_*th*_, 1.4*I*_*th*_, and 1.5*I*_*th*_. The ATP cost was, respectively, computed in the soma, yellow dendrite, and whole dendrites. **(C)** Left panel: time courses of somatic voltage *V*_*S*_ and dendritic voltage *V*_*D*_ at *I*_*syn*_ = 1.3*I*_*th*_. Right panel: time courses of *V*_*S*_ and *V*_*D*_ at *I*_*syn*_ = 1.4*I*_*th*_. The recording site of *V*_*S*_ and *V*_*D*_ were indicated by the triangles of same color in Figure 9A. *I*_*S*_ = 0 nA.

We also compared the ATP consumption on reversing Ca^2+^ influx in the dendrites with the consumption on Na^+^ ion pumping in the soma. The ATP requirements per unit area in the yellow dendrite and whole dendrites was both smaller as compared to the soma (Figure 10A). However, the total surface area of the whole dendrites was 21009.325065 μm^2^, which was much larger than somatic area (i.e., 1131.3891 μm^2^). Thus, more ATP molecules were required for reversing Ca^2+^ influx in the whole dendrites than for Na^+^ ion pumping in the soma (Figure 10B). At *I*_*syn*_ < 1.4*I*_*th*_, the Ca^2+^ spike only activates one Na^+^ spike in the soma (Figure 10C). At *I*_*syn*_ ≥ 1.4*I*_*th*_, two Na^+^ spikes were generated during a Ca^2+^ spike. Thus, the ATP cost in the soma at *I*_*syn*_ = 1.4*I*_*th*_ was about twofold more than that at *I*_*syn*_ = 1.3*I*_*th*_. This indicated that the generation of a Ca^2+^ AP made a significant contribution to the signaling-related energy in the pyramidal models, which was consistent with above simulations.

## Discussion

We used biologically realistic models of L5 pyramidal neurons to investigate the energy cost of a Ca^2+^ spike initiated in the apical dendrites. The energy cost was estimated with the following approaches. We integrated the inward Ca^2+^ currents to give the total Ca^2+^ influx, and then calculated the number of ATP molecules that were hydrolyzed to restore the homeostasis of intracellular Ca^2+^ concentrations, operating with a ratio of one Ca^2+^ ion per ATP. These calculations allowed us to develop a qualitative understanding of the effects of synaptic inputs, dendritic voltage, bAP, and Ca^2+^ inactivation on the energy consumption associated with a Ca^2+^ spike.

Energy budgets for neural computation indicated that the synaptic transmission mediated by pre- and postsynaptic mechanisms consumed about 55% of the total ATP used in neocortex cells (Attwell and Laughlin, 2001; Harris et al., 2012; Howarth et al., 2012). In particular, up to 50% signaling energy was expended on the actions of postsynaptic excitatory receptors in cortex neurons, such as, reversing ionic fluxes producing excitatory postsynaptic currents and dendritic APs. The Ca^2+^ spike is a typical suprathreshold response in the apical dendrites. Our simulations suggested that the Ca^2+^ spikes consumed substantial ATP molecules in the dendrites, which makes a significant contribution to the overall usage of signaling-related energy in pyramidal neurons. These data were consistent with earlier predictions of energy budgets in the cerebral cortex (Attwell and Laughlin, 2001; Howarth et al., 2012).

The single dendritic branch is a fundamental functional unit of signaling in the nervous system (Branco and Häusser, 2010). In L5 pyramidal cells, the dendrites receive a number of synaptic inputs from cortical layers and deliver them to the soma/axon. Each dendrite also acts as an independent processing and signaling unit, which performs supralinear and sublinear summations of the incoming signals as propagating them to the soma (Tran-Van-Minh et al., 2015). The dendritic integrations are crucial for the computational capability of a neuron. As a supralinear integration operated by the dendrites, the Ca^2+^ AP propagates distal signals to the soma/axon, which provides a cellular mechanism for coupling inputs arriving at different cortical layers (Larkum et al., 1999; Larkum, 2013). Our simulations indicated that the whole dendrites consumed substantial ATP on Ca^2+^ ion pumping during a Ca^2+^ spike. This high ATP demand may be related to the dendritic function of integrating signals.

Experimental recordings (Hallermann et al., 2012) made in the axon of pyramidal neurons reported that the metabolic cost and energy efficiency of a Na^+^ AP were dependent on the membrane potential. Our simulations showed that the dendritic voltage determined the inactivation and availability of voltage-gated Ca^2+^ channels. Thus, the ATP cost of a Ca^2+^ spike in L5 pyramidal model neurons also depended on dendritic voltage, which was consistent with the findings of Hallermann et al. (2012). The voltage dependence of Ca^2+^ spike cost in neocortical neurons makes it complicated to estimate the signaling-related energy with experimental approaches. In this sense, the computational modeling provides a powerful tool to understand the energy budget for signaling in a specific neuron.

Larkum et al. (1999) showed that the back-propagating Na^+^ spike in the soma/axon reduced the threshold of a Ca^2+^ spike and activated BAC firing in the apical dendrites when it coincided with synaptic inputs within a time window of several milliseconds (Schaefer et al., 2003; Larkum, 2013). We extended their results to show that the bAP regulated the ATP cost of a Ca^2+^ spike, which depended on the timing of synaptic inputs relative to the onset of bAP. Our results also indicated that the effects of a bAP on Ca^2+^ spike cost were due to its interactions with dendritic depolarization, which altered the availability of dendritic Ca^2+^ channels. Further, Larkum et al. (1999) also reported that the BAC firing was a cellular mechanism in pyramidal cells for coupling inputs arriving at different cortical layers. Based on our simulations, we inferred that the higher Ca^2+^ influx during a BAC firing may be for coupling cortical signals despite the additional metabolic cost.

Earlier studies predicted that Na^+^ inactivation governed the metabolic energy of a Na^+^ AP in the soma/axon (Hasenstaub et al., 2010; Hallermann et al., 2012). Fast Na^+^ inactivation increased Na^+^ entry efficiency and limited the ATP cost of Na^+^ spikes. On the contrary, slowing Na^+^ inactivation rate reduced the efficiency of Na^+^ influx, increasing spike cost. Our simulations were consistent with these previous studies, and we extended these prior observations to show that slowing Ca^2+^ inactivation increased the ATP cost of a dendritic Ca^2+^ spike, whereas accelerating Ca^2+^ inactivation reduced the Ca^2+^ spike cost. These data suggested that the inactivation kinetics of ion channels played considerable roles in the energy consumption of neuronal computation.

## Conclusion

To the best of our knowledge, this computational study was the first investigation of the metabolic energy associated with dendritic Ca^2+^ APs in pyramidal cells. Our simulations predicted that the ATP cost of a Ca^2+^ spike depended on the state of dendritic activity. Synaptic inputs, membrane voltage, bAP, and Ca^2+^ inactivation all contributed to determining the energy consumption of a dendritic Ca^2+^ spike. The results suggested that the pyramidal neurons were able to dynamically adjust their energy demand based on dendritic responses, which should be considered when understanding the dendritic processing in the pyramidal cells, the energy budget for signaling in the cerebral cortex, as well as the metabolism-dependent functional brain imaging signals. Based on computational modeling, we provided a qualitative interpretation of how subcellular processes and membrane biophysics were organized effectively and efficiently to perform neural computation. The predictions were important for inspiring engineers to design energy-efficient neuromorphic devices.

## Data Availability Statement

All datasets generated for this study are included in the article/supplementary material.

## Author Contributions

GY and JW conceived and designed the work, and wrote the manuscript. GY and YF performed the simulations. GY, YF, and JW analyzed and interpreted the data.

## Conflict of Interest

The authors declare that the research was conducted in the absence of any commercial or financial relationships that could be construed as a potential conflict of interest.
